# Education and Socio-economic status are key factors influencing use of insecticides and malaria knowledge in rural farmers in Southern Côte d’Ivoire

**DOI:** 10.1186/s12889-022-14446-5

**Published:** 2022-12-28

**Authors:** Ruth M. A. Kouamé, Federica Guglielmo, Kouabénan Abo, Allassane F. Ouattara, Joseph Chabi, Luigi Sedda, Martin J. Donnelly, Constant Edi

**Affiliations:** 1 Institut National Polytechnique Félix Houphouët Boigny, BP 1093 Yamoussoukro, Côte d’Ivoire; 2 Centre Suisse de Recherches Scientifiques en Côte d’Ivoire, 01 BP 1303, Abidjan, Côte d’Ivoire; 3 Liverpool School of Tropical Medicine, Vector Biology Department, Pembroke Place, Liverpool, L3 5QA United Kingdom; 4 Université Nangui Abrogoua, 02 BP 801 Abidjan, Côte d’Ivoire; 5 Noguchi Memorial Institute for Medical Research, Accra, Ghana; 6 Lancaster Ecology and Epidemiology Group, Lancaster Medical School, Lancaster University, Furness Building, Lancaster, LA1 4YG United Kingdom

**Keywords:** Insecticides, Mosquitoes, Socioeconomic status, Farmers, Malaria, Côte d’Ivoire

## Abstract

**Background:**

Insecticides play a key role in rural farming; however, their over- or misuse has been linked with a negative impact on malaria vector control policies. This study was conducted amongst agricultural communities in Southern Côte d’Ivoire to identify which insecticides are used by local farmers and how it relates to the perception of farmers on malaria. Understanding the use of insecticides may help in designing awareness programme on mosquito control and pesticides management.

**Methods:**

A questionnaire was administered to 1399 farming households across ten villages. Farmers were interviewed on their education, farming practices (e.g. crops cultivated, insecticides use), perception of malaria, and the different domestic strategies of mosquito control they use. Based on some pre-defined household assets, the socioeconomic status (SES) of each household was estimated. Statistical associations were calculated between different variables, showing significant risk factors.

**Results:**

The educational level of farmers was significantly associated with their SES (*p* < 0.0001). Most of the householders (88.82%) identified mosquitoes as the principal cause of malaria, with good knowledge of malaria resulting as positively related to high educational level (OR = 2.04; 95%CI: 1.35, 3.10). The use of indoor chemical compounds was strongly associated to the SES of the households, their education level, their use of ITNs and insecticide in agricultural (*p* < 0.0001). Indoor application of pyrethroid insecticides was found to be widespread among farmers as well as the use of such insecticide for crops protection.

**Conclusion:**

Our study shows that the education level remains the key factor influencing the use of insecticides by farmers and their awareness of malaria control. We suggest that better communication tailored to education level and including SES, controlled availability and access to chemical products, should be considered when designing campaigns on use of pesticides and vector borne disease control for local communities.

**Supplementary Information:**

The online version contains supplementary material available at 10.1186/s12889-022-14446-5.

## Introduction

Agriculture represents the key economic driver in many West African countries. In 2018 and 2019, Côte d’Ivoire was the world’s leading producer of cocoa and cashew nuts and the third African producer of coffee [1], with agricultural services and products representing 22% of the gross domestic product (GDP) [2]. Rural smallholders are the main contributors to the economic development of this sector as owners of most farming lands [3]. The country has considerable agricultural potential with 17 million ha of agricultural land and seasonal variation, which facilitate crop diversification and the cultivation of coffee, cocoa, cashew, rubber, cotton, yam, palm, cassava, rice, vegetables [2]. Intensified agriculture favours the proliferation of pests, controlled mainly through increased application of pesticides [4] especially among rural farmers in order to protect crops and increase productions [5], and to control mosquitoes [6]. However, inappropriate insecticide use is among the leading causes of insecticide resistance in disease vectors, especially in agricultural areas where mosquitoes and crop pests may be subject to selection pressure from the same insecticides [7–10]. The use of insecticides requires attention as a factor that could impact vector control strategies and environment by polluting [11–15].

Pesticides use among farmers has been investigated in the past [5, 16]. Education level has been shown to be a key factor in the correct use of insecticides [17, 18], although farmers pesticide use tend to rely on empirical experience or on the advice of retailers [5, 19, 20]. Financial difficulties are among the most common barriers constricting access to insecticides or pesticides, steering farmers towards banned or obsolete products, often found at lower prices than legal products [21, 22]. Similar trends have been reported in other West African countries, where low income was a reason for buying and using inappropriate pesticides [23, 24].

In Côte d’Ivoire, there is a widespread use of pesticides in crops [25, 26], which influences agricultural practices and malaria vector population [27–30]. Studies in malaria-endemic regions, have shown a relation between socioeconomic position and perceptions of malaria and risks of infection, as well as the use of insecticide-treated nets (ITNs) [31–37]. Despite these studies, efforts to establish concrete policies towards the management of mosquitoes are undermined by a lack of information about insecticides use in rural areas and the factors that could promote their appropriate use. This study investigates the perception of malaria and the strategies used to control mosquitoes among agricultural households from Agboville, Southern Côte d’Ivoire.

## Methods

### Description of the study area

**Fig. 1:**
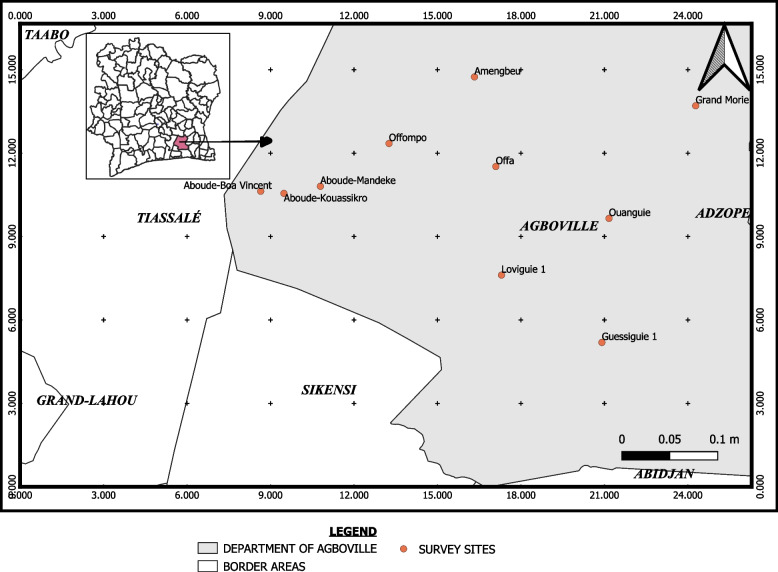
Localities surveyed in the department of Agboville

The study was carried out in ten villages of the department of Agboville in Southern Côte d’Ivoire (Fig. 1). The department of Agboville has a population of 292,109 inhabitants within 3,850 km^2^ and it is the most populated department of the Agnéby-Tiassa region [38]. The climate is tropical with two rainy seasons (April to July and October to November) [39, 40]. Farming is the main activity in the area, and it is conducted by smallholders and large agro-industrial firms. The 10 sites included Aboude Boa Vincent (323,729.62 E, 651,821.62 N), Aboude Kouassikro (326,413.09 E, 651,573.06 N), Aboude Mandeke (330,633.05 E, 652,372.90 N), Amengbeu (348,477.76 E, 664,971.70 N), Grand Morie (374,039.75 E, 661,579.59 N), Guessiguié 1 (363,140.15 E, 634,256.47 N), Loviguie 1 (351,545.32 E, 642,062.37 N), Offa (350,924.31 E, 654,607.17 N), Offompo (338,578.51 E, 657,302.17 N) and Ouanguie (363,990.74 E, 648,587.44 N).

### Study design and data collection

The study was carried out between August 2018 and March 2019 and involved agricultural households. The total number of inhabitants per village was obtained from the local services, and 1,500 were randomly selected from this list. Participants recruited represented between 6 and 16% of the village population. Households included in the study were those belonging to farmers who gave their consent to participate. A pre-questionnaire was conducted among 20 farmers to evaluate if there was a need to rewrite some questions. Then the questionnaire was conducted by trained and paid data collectors in each village, with at least one recruited from the village itself. This choice ensured that at least one data collector in each village was familiar with the environment and spoke the local language. In each household, a face-to-face interview was conducted with the household head (father or mother) or, in case of their absence, with another adult aged above 18. The questionnaire contained 36 questions, structured into three parts: (1) demographic and socioeconomic background of the household; (2) agricultural practices and pesticide use; and (3) knowledge of malaria and use of insecticide against mosquitoes [see Additional file 1].

### Data analysis

Pesticides mentioned by farmers were coded by their commercial names and sorted by active ingredients and chemical families using the Ivorian phytosanitary index [41]. Socioeconomic status of each household was assessed by calculating asset index [42]. Households assets were transformed into dichotomous variables [43]. A negative factor score was associated with lower socioeconomic status (SES) while a positive one to higher SES. The asset scores were summed to a total score for each household [35]. According to their total scores, the households were categorized into five SES quintiles, from the poorest to the wealthiest [see Additional file 4].

To determine whether a variable varied significantly across SES, villages or educational level of the household head, either Chi square or Fisher’s exact tests was used as appropriate. Logistics regressions models were fitted with the following predictors: education level, SES (both converted into dichotomous variables), villages (including as categorical variable), high knowledge of malaria and agricultural insecticide use, with as output the indoor insecticide use (by spray can or coil); education level, SES and villages, with as output the high knowledge of malaria. This logistic mixed regression model was performed using R package lme4 (glmer function). Statistical analyses were conducted in R 4.1.3 (https://www.r-project.org) and Stata 16.0 (StataCorp, College Station, Texas).

## Results

### Sociodemographic characteristics of household heads

Out of the 1,500 interviews conducted, 101 were excluded from analysis due to the uncompleted questionnaire. The highest proportion of interviewed households was in Grand Morie (18.87%) and the lowest in Ouanguie (2.29%). The 1399 interviewed households included in the analysis represented a population of 9,023 people. Household heads were 91.71% males and 8.29% females as shown in Table 1.

**Table 1:** Characteristic of household heads

Variables	Number	Frequency (%) [CI]^a^
*Sex*		
Male	1283	91.71 [90.14–93.10]
Female	116	8.29 [6.89–9.86]
*Educational level*		
Illiterate	248	17.97 [15.98–20.10]
Koranic school	44	3.19 [2.33–4.26]
Primary	563	40.80 [38.19–43.44]
Secondary	461	33.41 [30.92–35.96]
University	64	4.64 [3.59–5.88]
*Presence of children* (Yes)	1276	91.21 [89.60–92.64]
*Profession*		
Only farming	1250	89.35 [87.61–90.91]
Farming + other activities	149	10.65 [9.08–12.39]

^a^CI = Confidence intervals

About 8.86% of household heads were from neighbouring countries such as Benin, Mali, Burkina Faso, and Ghana. The most represented ethnic groups were Abbey (60.26%), Malinké (10.01%), Krobou (5.29%) and Baoulé (4.72%). As expected by selecting agricultural households, farming was the only source of income for most of them (89.35%); cocoa crops were the most cultivated by households sampled. There were also vegetables, food market crops, rice, rubber, and plantain cultivation on relatively small land surfaces. The rest of household heads were traders, art professionals and fishermen (Table 1). A summary of the household characteristics by village is shown in an additional file [see Additional file 3].

Education category did not differ by gender (*p* = 0.4672). The respondents with elementary (primary) educational level were the majority (40.80%) followed by secondary education level (33.41%) and illiterate (17.97%). Only 4.64% have reached the university (Table 1). Among the 116 women interviewed, more than 75% have at least the primary level and the others had never gone to school. Education levels of farmers varied significantly between villages (Fisher’s exact test, *p* < 0.0001) and the household heads educational level significantly and positively associated with their SES (Fisher’s exact test *p* < 0.0001). In fact, the higher SES quintiles are characterised mostly by farmers with higher level of education; and conversely the lowest SES quintiles by illiterate farmers. According to their total score of assets, sampled households were ranked into five wealth quintiles, from the poorest (Q1) to the wealthiest (Q5), [see Additional file 4].

The marital status of household heads was significantly different between wealth classes (*p* < 0.0001), with 83.62% being monogamous and 16.38% polygynous (with up to three spouses). No significant difference was found between the wealth classes and the number of spouses.

### Knowledge of malaria, malaria treatments, and use of ITNs

Most of the interviewees (88.82%) identified mosquitoes among the causes of malaria. Only 1.65% answered that they did not know what the cause of malaria was. Other causes identified were drinking dirty water, exposure to sun, bad food, and fatigue (Table 2). At the village level, in Grand Morie, most of the households identified drinking dirty water to be the main cause of malaria (statistically different between villages, *p* < 0.0001). The two main identified symptoms of malaria were high body temperature (78.38%) and yellow eyes (72.07%). Farmers also mentioned vomiting, anaemia, and paleness (see Table 2 below).

**Table 2:** Household head perception on malaria knowledge, prevention and treatment related to their SES

		**Poorest**	**Very poor**	**Poor**	**Less poor**	**Wealthiest**
	Total N (%)	*N = *298	*N = *298	*N* = 252	*N* = 273	*N* = 278
Malaria causes*						
**Mosquitoes**	699 (88.82)	80.1	89.89	88.72	96.58	92.44
**Dirty water**	375 (47.65)	15.42	57.98	50.38	72.6	52.1
**Sun**	251 (31.89)	23.38	31.38	33.83	42.47	31.93
**Bad food**	43 (5.46)	1.49	5.32	6.02	9.59	6.72
**Fatigue**	41 (5.21)	5.97	5.85	5.26	4.79	3.36
**Unknow**	13 (1.65)	2.99	0.53	2.26	0	2.52
Malaria symptoms*						
**Hot body**	609 (78.38)	60.71	79.14	78.63	86.9	95.76
**Vomiting**	478 (61.52)	58.67	63.64	55.73	62.76	67.8
**Pale body**	378 (48.65)	33.16	53.48	46.56	53.79	62.71
**Yellow eyes**	560 (72.07)	39.8	79.14	76.34	87.59	90.68
**Yellow urine**	505 (64.99)	29.08	74.87	63.36	80.69	91.53
**Anaemia**	407 (52.38)	30.1	56.68	48.85	61.38	75.42
***Malaria frequency on adults***						
**Rarely**	875 (63.64)	68.28	65.65	63.01	57.56	63.14
**Often**	410 (29.82)	22.76	28.57	31.3	34.69	32.48
**Very often**	90 (6.55)	8.97	5.78	5.69	7.75	4.38
***Malaria frequency on children***						
**Rarely**	118 (8.75)	6.79	9.44	6.22	5.95	15.07
**Often**	568 (42.14)	42.14	43.01	45.23	43.12	37.5
**Very often**	662 (49.11)	51.07	47.55	48.55	50.93	47.43
***Way of treatment***						
**Traditional**	34 (2.52)	3.94	2.06	3.28	2.23	1.12
**Modern**	236 (17.47)	11.47	12.71	15.16	21.93	26.49
**Both**	1081 (80.01)	84.59	85.22	81.56	75.84	72.39
***Cost of treatment***						
** < 10 000**	107 (7.76)	6.19	9.46	4.49	5.17	13.04
**10 000–30 000**	302 (21.9)	18.56	22.64	20.82	22.14	25.36
** > 30 000**	572 (41.48)	45.02	36.15	44.9	47.6	34.42
**Unknow**	398 (28.86)	30.24	31.76	29.8	25.09	27.17
***Bed net usage***						
	1192 (85.2)	77.85	85.23	84.92	89.38	89.21
***Insecticide use in crops***						
	1103 (78.84)	64.09	80.54	79.76	79.49	91.37
***Insecticide use in houses***						
	507 (36.24)	15.1	36.91	34.52	46.15	50.00
***Weekly insecticide use in houses***						
** < 3 times**	160 (32.72)	41.46	43.93	26.51	26.23	30.88
**3–4 times**	290 (59.3)	43.9	47.66	67.47	66.39	61.76
** > 4 times**	39 (7.98)	14.63	8.41	6.02	7.38	7.35

^*^Farmers reported more than one variable

Among prevention strategies against malaria, interviewees mentioned the use of traditional medicines; however, in case of illness, both biomedical and traditional approaches to treat malaria were mentioned as viable options (80.01%), with preferences significantly associated to SES (*p* < 0.0001): farmers with higher SES preferred, and were able to afford, biomedical treatment; at a lower SES, famers leaned towards more traditional, herbal treatments. The mean yearly money spent by almost half of households to treat malaria was more than 30,000 XOF (negatively associated with SES; *p* < 0.0001). According to self-reported estimates of direct expenses, households with the lowest SES were more likely to spend up to 30,000 XOF (around 50 US dollars) more for malaria treatment than household with the highest SES. Furthermore, most of the respondents perceived malaria to occur more often in children (49.11%) than in adults (6.55%) (Table 2), a perception more frequent among households belonging to the poorest quintiles (*p* < 0.01).

Against mosquito bites, most participant (85.20%) reported using ITNs, which they largely received during a national distribution occurred in 2017. Both adults and children were reported sleeping under ITNs in 90.99% of the households. In all the villages, the frequency of households with ITNs use was higher than 70%, except the village of Guessiguié where only 40% households reported to use them. The mean number of ITNs owned by households was significantly and positively associated to the dimensions of the household (Pearson’s correlation *r *= 0.41, *p* < 0.0001). Our findings also show that households with children under 1 year old are more likely to use ITNs in the house compared to households with none or older children (Odds Ratio (OR) = 2.08, 95%CI: 1.25–3.47).

### Farmers insecticide uses

Apart from ITNs use, farmers were asked about other means for mosquito control in their houses and about agricultural products used against crops pests. Only 36.24% of the participants mentioned spraying insecticides in their houses (significantly and positively associated to SES *p* < 0.0001). The chemical compounds reported belonged to nine commercials brand, mostly provided by the local market and some retailers in the form of fumigating coils (16.10%) and insecticides sprays (83.90%). The ability of farmers ability to provide the name of insecticides sprayed in their houses increased with their educational level (12.43%; *p* < 0.05). The agrochemical products in use were bought originally in cans and diluted in sprayers before application, with the highest proportion being generally aimed at crops (78.84%) (Table 2). The village of Amangbeu recorded the lowest proportions of farmers that use insecticides both in houses (0.93%) and in crops (16.67%).

The maximum number of declared insecticidal products per house, as spray or coils, was three, with a positive association between SES and the number of products used (Fisher exact test *p* < 0.0001); however, in some cases, such products were found to have the same active ingredients under different commercial names. Table 2 shows the frequency of weekly insecticides applied by farmers according to their SES.

**Fig. 2:**
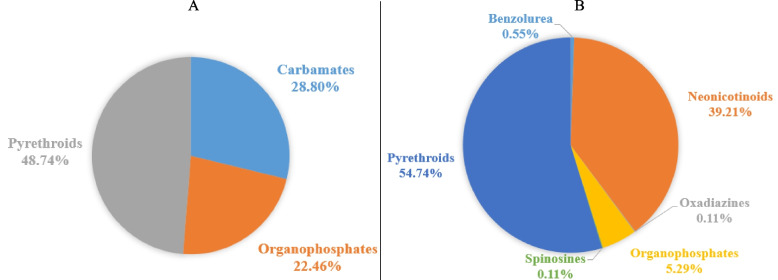
Chemical families of insecticides applied by farmers in their houses (**A**) and in their crops **B**

Pyrethroids were the most represented chemical family among both domestic (48.74%) and agricultural (54.74%) insecticides sprays. Products were made of each insecticide or in combination with other insecticides. The common combination in domestic insecticides were made of Carbamate, Organophosphate and Pyrethroid whereas in agricultural insecticide, Neonicotinoid and Pyrethroid were prevalent (Additional file 5). Figure 2 shows the proportion of the different insecticide families used by farmers, all of which were identified as belonging to class II (moderately hazardous) or class III (slightly hazardous) according to the WHO classification of pesticides [44]. On one occasion Deltamethrin insecticide designed for agricultural purposes was found to be used domestically.

Regarding active ingredients, Propoxur and Deltamethrin were the most common products intended for domestic and field use respectively. Details about the chemical products used by farmers both in their houses and in their crops are reported in Additional file 5.

Farmers mentioned other means of mosquito control, including fans made of leaves (*pêpê* in local language Abbey), burning some leaves, cleaning the surroundings, removing all stagnant water, using mosquito repellents, or simply chasing mosquitoes away with bed clothes.

Factors associated with farmers knowledge of malaria and indoor insecticides spray (logistic regression analyses).

**Fig. 3:**
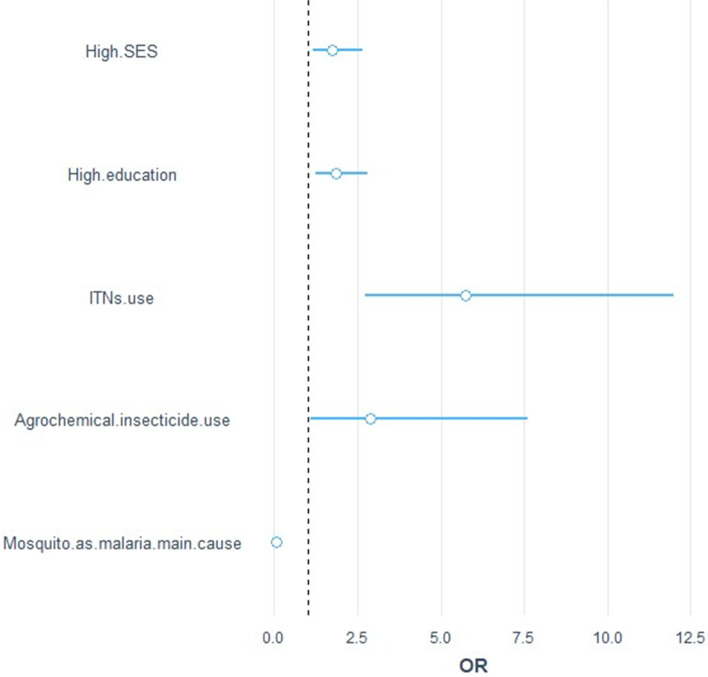
Odds Ratio (OR) of the five predictors of the indoor insecticide use

Data showed significative associations between indoor insecticide spray use and the five predictors: education level, SES, knowledge of mosquitoes as the main cause of malaria, use of ITN, and agrochemical insecticide use. The Fig. 3 shows the different OR of each predictor. When clustering between villages, all the predictors showed positive association with insecticide spray use in houses (except knowledge of the main cause of malaria, which was negatively associated with insecticide use (OR = 0.07, 95%CI: 0.03, 0.13)) (Fig. 3). Among these positive predictors, agricultural insecticides use represents an interesting factor. Farmers who applied insecticides to their crops were 188% more likely to apply insecticides in their houses (95%CI: 1.12, 8.26). However, insecticide use in houses was less likely in those households with high knowledge of malaria transmission. Knowledge of mosquitoes as the main cause of malaria was more likely in high education level groups (OR = 2.04; 95%CI: 1.35, 3.10) but not statistically associated with high SES (OR = 1.51; 95%CI: 0.93, 2.46).

### Perception on insecticides effect on mosquito behaviour

According to the household heads, mosquito abundance peaks during the wet season, citing the night as the time of most frequent bites (85.79%). As farmers were questioned about their perception of the effect of insecticides spraying on mosquito malaria vector population, 86.59% affirmed that mosquitoes seemed to become resistant to insecticides. The inability to use enough chemical product(s) due to unaffordable costs was indicated as the main contributor to insecticide resistance; product inefficacy or misuse were identified as other determinant factors. The latter, specifically, was associated with lower educational status (*p* < 0.01) even when controlling for SES (*p* < 0.0001). Mosquito robustness was identified as one possible reason of insecticide resistance by only 12.41% of interviewees.

There was a positive association between the frequency of insecticide used in the households and the perception on insecticide resistance in mosquitoes (*p* < 0.0001): insecticide resistance in mosquitoes was reported mostly by farmers who used insecticides in their houses 3 to 4 times per week (90.34%). Apart from the frequency, the number of insecticides used was also positively associated to insecticide resistance perception in farmers (*p* < 0.0001).

## Discussion

This study focused on perceptions of malaria and uses of insecticides among farmers. Our findings show that both education and SES play a key role in behavioural habits and knowledge of malaria. Although most of the household heads attended primary schools, the proportion of farmers with no education was considerable, as found elsewhere [35, 45]. This observation could be explained by the fact that even if many farmers began education, most of them had to leave school to support the family through agricultural activities [26]. Conversely, this phenomenon highlights the connection between SES and education as essential to explain the link between SES and the ability to act upon the information received.

### Household heads perception of malaria

Malaria causes and symptoms were well known to the participants as in many malaria endemic regions [33, 46–49]. In general, awareness of children susceptibility to malaria was widespread [31, 34]. This awareness might derive from a combination of the susceptibility of children as well as of the intensity of symptoms shown in case of malaria [50, 51].

Participants reported spending an average annual amount of 30,000 XOF for malaria treatment—half of the guaranteed minimum wage per month recommended in Côte d’Ivoire—and this is an underestimation: many farmers rely on traditional medicines and the figure addresses direct costs only, leaving aside factors such as loss of productivity, transport etc.

A comparison between farmers SES showed that farmers with the lowest SES reported spending more money than the wealthiest one. This might be due to the lowest SES households perceiving the expense as higher—since higher is its weight on the overall household finances—or on the collateral benefits of being employed in the public and private sector, as it was the case of wealthier households: thanks to health insurance, the money spent on malaria treatment (versus the overall cost) might be considerably inferior than the amount spent by households who do not benefit from insurance [52]. In fact, wealthiest households were reported to use mainly biomedical treatment when compared to poorest ones.

### ITNs and insecticide use

Even if most farmers identified mosquitoes as the principal cause of malaria, only few of them used insecticides in their houses (by spraying and fumigant coils), similarly to findings from Cameroon and Equatorial Guinea [48, 53]. This lack of attention to mosquitoes compared to crop pests is due to the economic values of the crops. To limit expenditures, low-cost method like burning leaves in their houses or simply chasing mosquitoes manually were preferred. Perceived toxicity is likely to be a factor too: the smell of some chemical products and bad experiences after their usage led some users avoiding them [54]. The high presence (with 85.20% reporting use) of ITNs in the households further motivates the low use of insecticides against mosquitoes. The presence of ITNs in households was also highly correlated with the presence of children under 1 years old, possibly due to ante-natal clinic support whereby pregnant women receive ITNs during prenatal consultation [6].

Pyrethroids being the main class of insecticides used both on ITNs [55] and by farmers for pests and mosquito control raises concerns on the surge of insecticide resistance [55–59]. This situation could explain the reduction of mosquito susceptibility to insecticides observed by farmers.

### SES and education related to farmers knowledge of malaria and attitudes toward insecticides

A higher SES was not related to better knowledge of malaria and of mosquitoes as its cause. Unlike previous findings of Ouattara and colleagues in 2011 showing that wealthier people tend to better identify malaria causes because of their easy access to information via TVs and radio [35]. Our analyses revealed that higher education level was the predictor which influence better knowledge of malaria. This observation confirms that education remains a key element in knowledge of malaria in farmers. The low impact of SES could be explained by the fact that sharing TVs and radio in village are not uncommon. However, SES should be considered when implementing knowledge in terms of domestic strategies of malaria avoidance.

Higher SES and higher education level was positively associated with domestic insecticide use (spray, or coil). Surprisingly, the ability of farmers to identify mosquitoes as the main cause of malaria had a negative influence in the model. When taking the overall population, this predictor was positively associated with insecticide use but after clustering between villages, it was negatively associated with insecticide use. This result shows the importance of anthropophagic effect on people behaviour and the need to consider include the random effect in the analysis. Our study is the first which shows that farmers with experience of agricultural insecticide use are most inclined than others to use sprays and coils insecticides as a domestic strategy against malaria.

Echoing previous work on SES influence on the attitudes of farmers towards pesticides [16, 60–63], wealthier households reported higher variability and frequency of insecticides use. Interviewees considered spraying large amounts of insecticide the best mean to avoid mosquito resistance, in line with concerns highlighted elsewhere [64]. Along these lines, farmers used domestic products that displayed the same chemical profile under different commercial names, which suggests that farmers technical knowledge of the products and their active ingredients should be prioritised. Attention should also be paid to retailers’ knowledge, as they are one of the main point of reference of insecticide buyers [17, 24, 65–67].

To positively affect insecticide, use in rural communities, policies and interventions should focus on improving communication strategies, considering educational level and behavioural habits within cultural and contextual adaptations, and on making safe insecticides accessible. People will buy according to the cost (how much they can afford) and the quality of the product. Once quality is offered at an acceptable cost, the need for behaviour change in buying good product is expected to be greatly improved; educating farmers on insecticides alternation to break down the chain of insecticide resistance, clarifying that alternation does not mean change in product brand (since different brands have the same active compound) but in the active ingredient. This education could be also supported by better labelling on products through easy and comprehensible representations.

## Conclusion

As pesticides are widespread used among rural farmers in the department of Agboville, understanding knowledge gaps and attitude of farmers towards the use of insecticides in their environment, appears as a prerequisite for designing successful awareness programmes. Our study confirms that education remains the main element for a correct use of insecticides and knowledge of malaria. Household socioeconomic status was identified to be an important tool to be considered as well. Apart from household heads SES and their educational level, other patterns like the knowledge of malaria, insecticide use for agricultural pest management, and perception of insecticide resistance in mosquitoes were found to influence farmers attitude towards insecticide use.

### Limitations

Respondent-dependent methods such as questionnaires are vulnerable to recollection and social desirability bias. Using household characteristic for assessing SES is relatively easy although these indicators may be specific to the temporal and geographical context in which they were developed and unevenly capture contemporary realities of culture-specific items of value, making comparison across studies difficult. In fact, there may be significant changes in household ownership of the index components, which may not necessarily translate into a reduction in material poverty.

Some farmers did not remember the insecticide product’s name, therefore the number of pesticides used by farmers can be under- or overestimated. Our study did not consider farmers attitudes when spraying pesticides and their perceptions of the consequences of their behaviours on their health and environments. Retailers also were not included in the study. Both points may be investigated in future research.

## Supplementary Information

**Additional file 1.** Questionnaire form.

**Additional file 2**. Information sheet for volunteer householders in the socio-economic survey.

**Additional file 3.** Farmers household characteristics among the ten localities sampled, frequency (percentage).

**Additional file 4.** Variables used in principal component analysis for describing index of wealth of each household.

**Additional file 5.** Active ingredients and chemical classes of insecticides used by farmers’ crops and houses.

## Data Availability

The datasets used and/or analysed during the current study are available from the corresponding author on reasonable request.

## Abbreviations

CI: Confidence Intervals
ITNs: Insecticidal Treated Nets
OR: Odds Ratio
SES: Socioeconomic status
WHO: World Health Organization
