# Supplementary material for: Education and Socio-economic status are key factors influencing use of insecticides and malaria knowledge in rural farmers in Southern Côte d’Ivoire
Source: BMC Public Health. 2022 Dec 28;22:2443. doi: 10.1186/s12889-022-14446-5 (PMC9795670; doi:10.1186/s12889-022-14446-5)
Supplement: Supplementary file 1 — Additional file 1. Questionnaire form. [file 12889_2022_14446_MOESM1_ESM.docx]

**Additional file 1.** Questionnaire form

Interviewer Name: ……...………………….….……….Village:……………………….Date: … / ….. / 201..

1. **Interviewee identification**

- Gender: Male Female
- Name: ……………………. Surname: ………………..…….………………………Contact:..…………
- Ethnic group: Abbey Abidji Attié Baoulé

Malinké Krobou other (specify): ……………………

- Affiliation with the village: Native Non-native
- Matrimonial status: Single Married Divorced Widower Widow
- If married: Monogamous Polygamous If polygamous, indicate the number…….
- Number of children: ……how many have: Above 5 years Between 1 – 5 years Under 1 year
- Number of dependent children: …………..…… Number of Schoolchildren:..………….……
- Religion: Christian Muslim Animist Atheist

other (specify): …..…….………………………………………………………

- Education: Primary Secondary University Islamic school Illiterate
- Occupation: Farmer Fisherman/breeder Trader Artisan Teacher

other (specify): …………………………………………………………………………....

1. **Household living standard**

- House wall type: Cement Bamboo/Wood Traditional terra cotta

Other (specify)……….……………………………………………………...

- House roof type: Metal Wood/Straw Plastic sheet

Other (specify)………..…………………………………………...

- Water supply: Yes No
- Electricity: Yes No
- Cooking energy: Gas Coal Wood Traditional fireplace

Other (specify)……….……..…………………………………………...

- Comfort: Radio Television CD DVD Refrigerator

Bicycle Motorcycle Telephone Ventilator

1. **Agricultural crops insects’ management**

- Crop cultivated

| Name of the culture | Land area  (to be ticked) | | | Pesticide use* |
| --- | --- | --- | --- | --- |
|  | < 1ha | 1–10 ha | > 10 ha |  |
|  |  |  |  |  |
|  |  |  |  |  |
|  |  |  |  |  |
|  |  |  |  |  |
|  |  |  |  |  |
|  |  |  |  |  |
|  |  |  |  |  |

*1 = Yes; 2= No; 3= Don’t know.

- Insecticide use in agricultural crops

| Products names | Target | Dosage  (l/ha) | Periods*  (P, C, B) | Frequency*  (A, B, C, D) |
| --- | --- | --- | --- | --- |
|  |  |  |  |  |

*P=Preventive; C=Curative; B=Both

*A= 1-2 times/cycle; B= 3-5 times/cycle; C= more than 5 times/cycle; D= don’t know.

**4. Knowledge of malaria and mosquitoes’ management**

- Cause of malaria

Sun Mosquitoes Fatigue Bad food Dirty water Don’t know

Other (specify) …………………………………………………………………………………………

- Malaria symptom

Hot body Vomiting Pale body Yellow eyes Yellow urine Anaemia

Don’t know

Other (specify) …………………………………………………………………………………………

- Malaria case frequency in the family:

Adult Rarely (1-2 times) Often (3-4 times) Very often (+ de 5 times) Don’t know

Children Rarely (1-2 times) Often (3-4 times) Very often (+ de 5 times) Don’t know

- Treatment: Traditional medicines Modern medicines Both
- Spending on malaria treatment per year:

Less than 10 000 FCFA 10 000-30 000 FCFA More than 30 000 FCFA Don’t know

- Presence of gutters near the house: Yes No Don’t know
- Presence of mosquitoes in the house: Yes No Don’t know
- If yes, period of mosquitoes’ bites: At day At night Both Don’t know
- Are they noisy mosquitoes? Yes No Don’t know
- Season of mosquitoes’ abundance: Dry season Rainy season Don’t know
- Other descriptive elements of mosquitoes:………………………………………………………………..

…………………………………………………………………………………………………………………

- Bednets use: Yes No Don’t know
- If yes, indicate the number…………………… and the year of acquisition ………………….
- Person sleeping under bednets: Adults Children Both
- Insecticides use against mosquitoes: Yes No Don’t know

If yes, indicate insecticides names……………………………………………………………………..

If yes, indicate the provider…………………………………………………………………………

- Insecticides frequency use:

Less than 3 times/week 3-4 times/week More than 4 times/week Don’t know

- Alternative controls against mosquitoes: Yes No Don’t know

If yes, indicate them…………………………………………………………………………………………….

- Observation of mosquitoes’ resistance to insecticides: Yes No Don’t know
- According to you, why these mosquitoes don’t die although control methods used?

Inefficiency of the product Misuse of the product Robustness of the insect

Lack of money to buy product in a sufficient quantity Don’t know

Other (specify) …………………………………………………………………………………………
