# Supplementary material for: Education and Socio-economic status are key factors influencing use of insecticides and malaria knowledge in rural farmers in Southern Côte d’Ivoire
Source: BMC Public Health. 2022 Dec 28;22:2443. doi: 10.1186/s12889-022-14446-5 (PMC9795670; doi:10.1186/s12889-022-14446-5)
Supplement: Supplementary file 2 — Additional file 2. Information sheet for volunteer householders in the socio-economic survey. [file 12889_2022_14446_MOESM2_ESM.pdf]

Additional file 2. Information sheet for volunteer householders in the socio-economic survey.

Introduction:

I, the undersigned, Dr. EDI Constant, Senior Researcher at the Centre Suisse de Recherches Scientifiques in Côte d'Ivoire (CSRS) and principal investigator of the project, invite you to read this document before accepting to participate in the project. This study consists in assessing the knowledge of households on malaria and their use of insecticides in the Agneby-Tiassa region of Côte d'Ivoire.

Study context: The National Malaria Control Program (NMCP) has implemented several vector control programs in recent years to reduce the incidence and burden of malaria. The success of these interventions is based on the effectiveness of the use of these control methods by the population. This can be influenced by different factors such as the level of education of the population, their level of knowledge about malaria and their socio-economic position. This is the framework in which our socio-economic study was carried out.

Procedure and type of intervention: We will ask a series of questions divided into 3 main parts to the households that are willing to take part in the survey. As a volunteer, you should answer the questions asked, but you can stop the survey at any time.

Risks: There is no risk for the interviewee in this study.

Benefits: By participating in this study, you will not benefit personally. Your participation is voluntary, but your participation will provide us with the information we need to conduct our study.

Right to refuse or withdraw: You are not obliged to participate in this study if you do not want to. If you choose to participate, you can stop your participation at any time without affecting your rights if you are ill.

Who to contact? If you have any questions now or in the future, you can contact **Dr Edi Constant (+225 0778701177)**. The President of the National Ethics and Research Committee: **Dr Louis Penali (+225 0707340707)**.

CERTIFICATE OF INFORMED CONSENT

The briefing note was read to me by the investigator. I have been given the opportunity to ask questions, and to all the questions I have asked, I have been given satisfactory answers. I am willing to volunteer as a participant in this study and I have the right to stop my participation at any time without losing any of my rights in case I become ill. I certify that I have received a copy of the informed consent this day.

Name of participant, Date and signature

________________ ___/___/___ (DD/MM/YY)

If illiterate,

I acknowledge that I witnessed the accurate reading of the informed consent information sheet to the participant who was given the opportunity to ask questions, and who was given satisfactory answers to any questions asked. I confirm that the participant has given consent freely.

Name of witness, Date and signature

________________ ___/___/___ (DD/MM/YY)

I have read or witnessed the accurate reading of the informed consent information sheet to the participant who was given the opportunity to ask questions, and who had satisfactory answers to any questions asked. I confirm that the participant has given consent freely.

Name of researcher, Date and signature

________________ ___/___/___ (DD/MM/YY)
