# Supplementary material for: Education and Socio-economic status are key factors influencing use of insecticides and malaria knowledge in rural farmers in Southern Côte d’Ivoire
Source: BMC Public Health. 2022 Dec 28;22:2443. doi: 10.1186/s12889-022-14446-5 (PMC9795670; doi:10.1186/s12889-022-14446-5)
Supplement: Supplementary file 3 — Additional file 3. Farmers household characteristics among the ten localities sampled, frequency (percentage). [file 12889_2022_14446_MOESM3_ESM.docx]

**Additional file 3.** Farmers household characteristics among the ten localities sampled, frequency (percentage).

|  | Aboude Boa Vincent | Aboude Kouassikro | Aboude Mandeke | Amengbeu | Grand Morie | Guessiguie 1 | Loviguie 1 | Offa | Offompo | Ouanguie | Total |
| --- | --- | --- | --- | --- | --- | --- | --- | --- | --- | --- | --- |
| Sex |  |  |  |  |  |  |  |  |  |  |  |
| Male | 93 (93.94) | 147 (96.08) | 73 (91.25) | 108 (100) | 230 (87.12) | 131 (99.24) | 235 (93.63) | 97 (72.93) | 140 (95.24) | 29 (90.63) | 1283 (91.71) |
| Female | 6 (6.06) | 6 (3.92) | 7 (8.75) | 0 (0) | 34 (12.88) | 1 (0.76) | 16 (6.37) | 36 (27.07) | 7 (4.76) | 3 (9.38) | 116 (8.29) |
| Total | 99 | 153 | 80 | 108 | 264 | 132 | 251 | 133 | 147 | 32 | 1399 |
| Marital status | |  |  |  |  |  |  |  |  |  |  |
| Single | 18 (18.18) | 19 (12.42) | 25 (31.25) | 19 (17.59) | 66 (25) | 109 (82.58) | 36 (14.34) | 17 (12.78) | 15 (10.2) | 23 (71.88) | 347 (24.8) |
| Married | 72 (72.73) | 131 (85.62) | 41 (51.25) | 80 (74.07) | 178 (67.42) | 16 (12.12) | 197 (78.49) | 104 (78.2) | 130 (88.44) | 5 (15.63) | 954 (68.19) |
| Divorced | 9 (9.09) | 2 (1.31) | 12 (15) | 6 (5.56) | 7 (2.65) | 3 (2.27) | 10 (3.98) | 9 (6.77) | 0 (0) | 1 (3.13) | 59 (4.22) |
| Widower | 0 (0) | 1 (0.65) | 2 (2.5) | 3 (2.78) | 13 (4.92) | 4 (3.03) | 8 (3.19) | 3 (2.26) | 2 (1.36) | 3 (9.38) | 39 (2.79) |
| Household with at least one child | | | |  |  |  |  |  |  |  |  |
|  | 89 (89.9) | 136 (88.89) | 61 (76.25) | 102 (94.44) | 261 (98.86) | 123 (93.18) | 222 (88.45) | 115 (86.47) | 138 (93.88) | 29 (90.63) | 1276 (91.21) |
| Household with child ages | | |  |  |  |  |  |  |  |  |  |
| > 5 years | 84 (84.85) | 120 (78.43) | 43 (53.75) | 98 (90.74) | 242 (91.67) | 118 (89.39) | 192 (76.49) | 89 (66.92) | 125 (85.03) | 26 (81.25) | 1137 (81.27) |
| 1-5 years | 43 (43.43) | 64 (41.83) | 25 (31.25) | 70 (64.81) | 60 (22.73) | 35 (26.52) | 137 (54.58) | 67 (50.38) | 86 (58.5) | 12 (37.5) | 599 (42.82) |
| < 1 year | 38 (38.38) | 34 (22.22) | 10 (12.5) | 13 (12.04) | 14 (5.3) | 3 (2.27) | 46 (18.33) | 13 (9.77) | 41 (27.89) | 3 (9.38) | 215 (15.37) |
| Education | |  |  |  |  |  |  |  |  |  |  |
| Illiterate | 39 (43.33) | 76 (50.67) | 17 (21.25) | 27 (25.47) | 29 (11.03) | 0 (0) | 5 (2) | 9 (6.82) | 39 (26.71) | 7 (21.88) | 248 (17.97) |
| Koranic school | 4 (4.44) | 20 (13.33) | 1 (1.25) | 0 (0) | 1 (0.38) | 0 (0) | 1 (0.4) | 4 (3.03) | 13 (8.9) | 0 (0) | 44 (3.19) |
| Primary | 25 (27.78) | 25 (16.67) | 23 (28.75) | 48 (45.28) | 127 (48.29) | 96 (73.28) | 119 (47.6) | 46 (34.85) | 38 (26.03) | 16 (50) | 563 (40.8) |
| Secondary | 16 (17.78) | 25 (16.67) | 26 (32.5) | 31 (29.25) | 82 (31.18) | 35 (26.72) | 120 (48) | 68 (51.52) | 50 (34.25) | 8 (25) | 461 (33.41) |
| University | 6 (6.67) | 4 (2.67) | 13 (16.25) | 0 (0) | 24 (9.13) | 0 (0) | 5 (2) | 5 (3.79) | 6 (4.11) | 1 (3.13) | 64 (4.64) |
| Religion |  |  |  |  |  |  |  |  |  |  |  |
| Animist | 4 (4.04) | 26 (16.99) | 3 (3.75) | 21 (19.44) | 34 (12.88) | 36 (27.27) | 7 (2.79) | 2 (1.5) | 4 (2.72) | 0 (0) | 137 (9.79) |
| Atheist | 2 (2.02) | 1 (0.65) | 2 (2.5) | 1 (0.93) | 30 (11.36) | 0 (0) | 13 (5.18) | 9 (6.77) | 7 (4.76) | 0 (0) | 65 (4.65) |
| Christian | 36 (36.36) | 50 (32.68) | 63 (78.75) | 78 (72.22) | 173 (65.53) | 95 (71.97) | 218 (86.85) | 103 (77.44) | 77 (52.38) | 31 (96.88) | 924 (66.05) |
| Muslim | 54 (54.55) | 73 (47.71) | 9 (11.25) | 5 (4.63) | 26 (9.85) | 0 (0) | 10 (3.98) | 17 (12.78) | 59 (40.14) | 0 (0) | 253 (18.08) |
| Other | 3 (3.03) | 3 (1.96) | 3 (3.75) | 3 (2.78) | 1 (0.38) | 1 (0.76) | 3 (1.2) | 2 (1.5) | 0 (0) | 1 (3.13) | 20 (1.43) |
| Occupation | |  |  |  |  |  |  |  |  |  |  |
| Only farmer | 97 (97.98) | 131 (85.62) | 64 (80) | 106 (98.15) | 196 (74.24) | 129 (97.73) | 246 (98.01) | 129 (96.99) | 120 (81.63) | 32 (100) | 1250 (89.35) |
| Trader | 2 (2.02) | 11 (7.19) | 6 (7.5) | 1 (0.93) | 23 (8.71) | 0 (0) | 2 (0.8) | 1 (0.75) | 11 (7.48) | 0 (0) | 57 (4.07) |
| Artisan, fisherman | 0 (0) | 6 (3.92) | 2 (2.5) | 0 (0) | 29 (10.98) | 1 (0.76) | 2 (0.8) | 1 (0.75) | 8 (5.44) | 0 (0) | 49 (3.5) |
| Other | 0 (0) | 5 (3.27) | 8 (10) | 1 (0.93) | 16 (6.06) | 2 (1.52) | 1 (0.4) | 2 (1.5) | 8 (5.44) | 0 (0) | 43 (3.07) |
