# Supplementary material for: Education and Socio-economic status are key factors influencing use of insecticides and malaria knowledge in rural farmers in Southern Côte d’Ivoire
Source: BMC Public Health. 2022 Dec 28;22:2443. doi: 10.1186/s12889-022-14446-5 (PMC9795670; doi:10.1186/s12889-022-14446-5)
Supplement: Supplementary file 4 — Additional file 4. Variables used in principal component analysis for describing index of wealth of each household. [file 12889_2022_14446_MOESM4_ESM.docx]

**Additional file 4**. Variables used in principal component analysis for describing index of wealth of each household.

|  |  |  | Poorest (Q1) | Very poor (Q2) | Poor (Q3) | Less poor (Q4) | Wealthiest (Q5) |
| --- | --- | --- | --- | --- | --- | --- | --- |
|  |  |  | (N=298) | (N=298) | (N=252) | (N=273) | (N=278) |
| Asset | **Mean** | **Factor score** |  |  |  |  |  |
| House type |  |  |  |  |  |  |  |
| Cement | 0.5014 | 0.2541 | 2 | 119 | 155 | 201 | 222 |
| Bamboo | 0.2597 | -0.1719 | 185 | 77 | 35 | 41 | 24 |
| Traditional | 0.2389 | -0.1212 | 109 | 99 | 62 | 31 | 32 |
| Roof type |  |  |  |  |  |  |  |
| Metal | 0.8059 | 0.2775 | 73 | 272 | 241 | 263 | 272 |
| Plastic | 0.1524 | -0.2628 | 193 | 10 | 4 | 4 | 1 |
| Wood | 0.0417 | -0.0765 | 32 | 12 | 5 | 4 | 5 |
| Water supply | 0.4652 | 0.1366 | 84 | 106 | 123 | 143 | 186 |
| Electricity | 0.8043 | 0.2255 | 179 | 239 | 197 | 234 | 265 |
| Cooking mode |  |  |  |  |  |  |  |
| Gas | 0.208 | 0.2965 | 4 | 2 | 9 | 80 | 196 |
| Wood fire | 0.8192 | -0.165 | 267 | 281 | 222 | 206 | 170 |
| Coal | 0.2495 | 0.2332 | 8 | 16 | 47 | 99 | 179 |
| Fireplace | 0.1337 | 0.0751 | 41 | 26 | 32 | 32 | 56 |
| Other commodities |  |  |  |  |  |  |  |
| Television | 0.4289 | 0.3186 | 12 | 31 | 111 | 190 | 256 |
| Radio | 0.5132 | 0.1407 | 75 | 135 | 145 | 164 | 199 |
| CD* | 0.1737 | 0.316 | 0 | 1 | 5 | 35 | 202 |
| DVD* | 0.1737 | 0.316 | 0 | 1 | 5 | 35 | 202 |
| Motorcycle | 0.2473 | 0.1948 | 9 | 22 | 54 | 110 | 151 |
| Bicycle | 0.5039 | 0.0633 | 105 | 156 | 137 | 141 | 166 |
| Fan | 0.3417 | 0.3052 | 4 | 31 | 55 | 151 | 237 |
| Freezer | 0.05218 | 0.1946 | 0 | 1 | 1 | 7 | 64 |
| Phone | 0.1959 | 0.0266 | 41 | 49 | 49 | 68 | 67 |

*CD=Compact Disc; DVD=Digital Versatile Disc.
