# Supplementary material for: Education and Socio-economic status are key factors influencing use of insecticides and malaria knowledge in rural farmers in Southern Côte d’Ivoire
Source: BMC Public Health. 2022 Dec 28;22:2443. doi: 10.1186/s12889-022-14446-5 (PMC9795670; doi:10.1186/s12889-022-14446-5)
Supplement: Supplementary file 5 — Additional file 5. Active ingredients and chemical classes of insecticides used by farmers’ crops and houses. [file 12889_2022_14446_MOESM5_ESM.docx]

**Additional file 5**. Active ingredients and chemical classes of insecticides used by farmers’ crops and houses

| In cultivated areas | Class | Active ingredient | Number of product |
| --- | --- | --- | --- |
|  | Neonicotinoid | Imidacloprid | 33 |
|  |  | Thiamethoxam | 84 |
|  | Neonicotinoid + Spinosine | Acetamiprid+spinetoram | 1 |
|  | Neonicotinoid+ Oxadiazine | Acetamiprid+indoxacarb | 1 |
|  | Neonicotinoid+Pyrethroid | Acetamiprid+bifenthrin | 12 |
|  |  | Acetamiprid+cypermethrin | 24 |
|  |  | Acetamiprid+lambdacyhalothrin | 61 |
|  |  | Imidacloprid+bifenthrin | 102 |
|  |  | Imidacloprid+cypermethrin | 1 |
|  |  | Imidacloprid+deltamethrin | 1 |
|  |  | Imidacloprid+lambdacyhalothrin | 13 |
|  |  | Thiacloprid+deltamethrin | 1 |
|  |  | Thiamethoxam+lambacyhalothrin | 22 |
|  | Organophosphate | Chlorpyriphos-ethyl | 7 |
|  |  | Profenofos | 2 |
|  | Pyrethroid | Bifenthrin | 2 |
|  |  | Cypermethrin | 41 |
|  |  | Deltamethrin | 156 |
|  |  | Lambdacyhalothrin | 17 |
|  | Pyrethroid + Benzoylurea | Bifenthrin+novaluron | 3 |
|  |  | Alphacypermethrin+teflubenzuron | 2 |
|  | Pyrethroid+ Organophosphate | Cypermethrin+profenofos | 39 |
| In houses | Carbamate | Propoxur | 78 |
|  | Carbamate+Organophosphate+Pyrethroid | Propoxur+dichlorvos+cyfluthrin | 276 |
|  | Pyrethroid | D-Phenothrin+ D-allethrin+ imiprothrin | 186 |
|  |  | D-Allethrin | 109 |
|  |  | Allethrin+deltamethrin+tetramethrin | 27 |
|  |  | Deltamethrin | 1 |
